# A novel lentiviral vector-based approach to generate chimeric antigen receptor T cells targeting *Aspergillus fumigatus*

**DOI:** 10.1128/mbio.03413-23

**Published:** 2024-02-28

**Authors:** Pappanaicken R. Kumaresan, Sebastian Wurster, Karishma Bavisi, Thiago Aparecido da Silva, Paul Hauser, Jordan Kinnitt, Nathaniel D. Albert, Uddalak Bharadwaj, Sattva Neelapu, Dimitrios P. Kontoyiannis

**Affiliations:** 1 Department of Hematopoietic Biology & Malignancy, The University of Texas MD Anderson Cancer Center, Houston, Texas, USA; 2 Department of Lymphoma & Myeloma, The University of Texas MD Anderson Cancer Center, Houston, Texas, USA; 3 Department of Infectious Diseases, Infection Control and Employee Health, The University of Texas MD Anderson Cancer Center, Houston, Texas, USA; 4 The International School of Pharmaceutical Sciences in Araraquara, Sao Paulo, Brazil; 5 Department of Pediatrics, The University of Texas MD Anderson Cancer Center, Houston, Texas, USA; IMBB-FORTH, Heraklion, Greece

**Keywords:** immunotherapy, aspergillosis, CAR T cells, T lymphocytes, cytokines, mouse model

## Abstract

Invasive aspergillosis (IA) is a common and deadly mold infection in immunocompromised patients. As morbidity and mortality of IA are primarily driven by poor immune defense, adjunct immunotherapies, such as chimeric antigen receptor (CAR) T cells, are direly needed. Here, we propose a novel approach to generate *Aspergillus fumigatus* (AF)-CAR T cells using the single-chain variable fragment domain of monoclonal antibody AF-269-5 and a lentiviral vector system. These cells successfully targeted mature hyphal filaments of representative clinical and reference AF isolates and elicited a potent release of cytotoxic effectors and type 1 T cell cytokines. Furthermore, AF-CAR T cells generated from peripheral blood mononuclear cells of four healthy human donors and expanded with either of three cytokine stimulation regimens (IL-2, IL-2 + IL-21, or IL-7 + IL-15) significantly suppressed mycelial growth of AF-293 after 18 hours of co-culture and synergized with the immunomodulatory antifungal agent caspofungin to control hyphal growth for 36 hours. Moreover, cyclophosphamide-immunosuppressed NSG mice with invasive pulmonary aspergillosis that received two doses of 5 million AF-CAR T cells (6 and 48 hours after AF infection) showed significantly reduced morbidity on day 4 post-infection (*P* < 0.001) and significantly improved 7-day survival (*P* = 0.049) compared with mice receiving non-targeting control T cells, even without concomitant antifungal chemotherapy. In conclusion, we developed a novel lentiviral strategy to obtain AF-CAR T cells with high targeting efficacy, yielding significant anti-AF activity *in vitro* and short-term protection *in vivo*. Our approach could serve as an important steppingstone for future clinical translation of antifungal CAR T-cell therapy after further refinement and thorough preclinical evaluation.

**IMPORTANCE:**

Invasive aspergillosis (IA) remains a formidable cause of morbidity and mortality in patients with hematologic malignancies and those undergoing hematopoietic stem cell transplantation. Despite the introduction of several new *Aspergillus*-active antifungals over the last 30 years, the persisting high mortality of IA in the setting of continuous and profound immunosuppression is a painful reminder of the major unmet need of effective antifungal immune enhancement therapies. The success of chimeric antigen receptor (CAR) T-cell therapy in cancer medicine has inspired researchers to translate this approach to opportunistic infections, including IA. Aiming to refine anti-*Aspergillus* CAR T-cell therapy and improve its feasibility for future clinical translation, we herein developed and validated a novel antibody-based CAR construct and lentiviral transduction method to accelerate the production of CAR T cells with high targeting efficacy against *Aspergillus fumigatus*. Our unique approach could provide a promising platform for future clinical translation of CAR T-cell-based antifungal immunotherapy.

## INTRODUCTION

Invasive fungal infections pose a significant threat to human life and are responsible for about 1.5 million deaths each year (1). Invasive aspergillosis (IA), particularly, invasive pulmonary aspergillosis (IPA), is the most common invasive mold infection in immunocompromised patients, including those with hematological malignancies (HM) and recipients of solid organ transplants or hematopoietic stem cell transplants (HSCT) (2). *Aspergillus fumigatus* (AF) is the most frequent cause of IA and is associated with significant morbidity, mortality, and health care burden (2).

Although severe neutropenia remains the predominant risk factor for IA and is associated with dismal prognosis of IA in patients with HM (3), several studies have also linked impaired T-cell-mediated immunity to poor IA outcomes (4, 5). Specifically, CD4^+^ T-helper (Th) cells are pivotal orchestrators of host immune defense against AF (6), and the establishment of effective antifungal Th responses is considered a significant prognostic factor in IA patients (4). Moreover, low concentrations of CD8^+^ cytotoxic T-lymphocytes were identified as a significant predictor of IA risk and mortality in critically ill patients (7).

Given the limitations of conventional antifungal therapy (e.g., toxicities, drug-drug interactions, and emerging antifungal drug resistance) and the prognostic significance of adaptive immune recovery, various T-cell-based immunotherapies against IA have been explored. Specifically, several groups proposed adoptive transfer of *Aspergillus*-specific T cells to restore antifungal host immunity (8, 9). Although yielding significant short-term benefits in IA patients (10), a critical drawback of adoptive T cells is their dependency on major histocompatibility complex (MHC)-expressed antigens. Hence, functional phagocytic responses are paramount for the effective deployment of this approach, thereby limiting the efficacy of adoptive T cells in IA patients with pancytopenia. To overcome this limitations, chimeric antigen receptor (CAR) T cells could present an attractive, MHC-independent approach.

CARs are engineered T-cell receptors that include an extracellular domain, most commonly, a single-chain variable fragment (scFv) of a monoclonal antibody (mAb), which recognizes the target antigen. The receptor is connected via a spacer and transmembrane domain to an intracellular signaling domain that activates downstream effector responses upon antigen binding (11). Thereby, CAR T-cell therapy combines the specificity of a mAb with the potent effector machinery of cytotoxic T cells (12).

The success of CAR T-cell therapy in cancer medicine (13) has inspired researchers to translate this approach to opportunistic infections. Specifically, three CARs have been engineered to target fungal cell wall components: a Dectin-1-CAR T cell to target β-glucans (14), a GXMR-CAR to target glucuronoxylomannan (GXM) in the cell wall of *Cryptococcus neoformans* (15), and an AF-CAR to target a thus far uncharacterized protein antigen present on AF hyphae (16).

The second-generation Dectin-1-CAR previously proposed by our group demonstrated specific recognition of β-glucans, elicited antifungal activity against AF *in vitro*, and conferred modest *in vivo* protection in immunocompromised mice with IA (14). However, Dectin-1 was shown to interact predominantly with early germlings of AF and has significantly diminished affinity to mature hyphae, presumably due to the masking of β-glucans by other cell wall components, such as glycosaminoglycans (17). Although a recently published AF-specific CAR T-cell product could overcome this limitation, there is an unmet need for streamlined production of these cells. Specifically, all published antifungal CAR T-cell products have relied on electroporation to incorporate the sleeping beauty transposon/transposase system for genomic insertion, a process that is often inefficient and can cause considerable cell death, therefore necessitating long culture periods (≥2 weeks) to obtain enough viable CAR T cells. As an alternative approach, viral vectors facilitate the transfer of genetic material with high efficiency, allow for more rapid production of CAR T cells, and have been essential for the development of current clinically approved oncological T-cell therapies (18).

Moreover, CAR T cells can elicit immune-related adverse events (irAEs), including cytokine release syndrome (CRS) and immune effector cell-associated neurotoxicity syndrome (19, 20). Notably, CAR T cells containing a CD28 costimulatory domain (21), which had been included in all previously published antifungal CAR T-cell designs, were reported to have a higher propensity to cause irAEs than constructs with a CD137/4-1BB costimulatory domain (22, 23).

Aiming to improve the feasibility of antifungal CAR T-cell therapy, we herein developed a novel, antibody-based AF-CAR with a CD137/4-1BB costimulatory domain and generated AF-CAR T cells using a rapid lentiviral (LV) expression strategy.

## RESULTS

### Production of mAb AF-269-5

**Fig 1:**
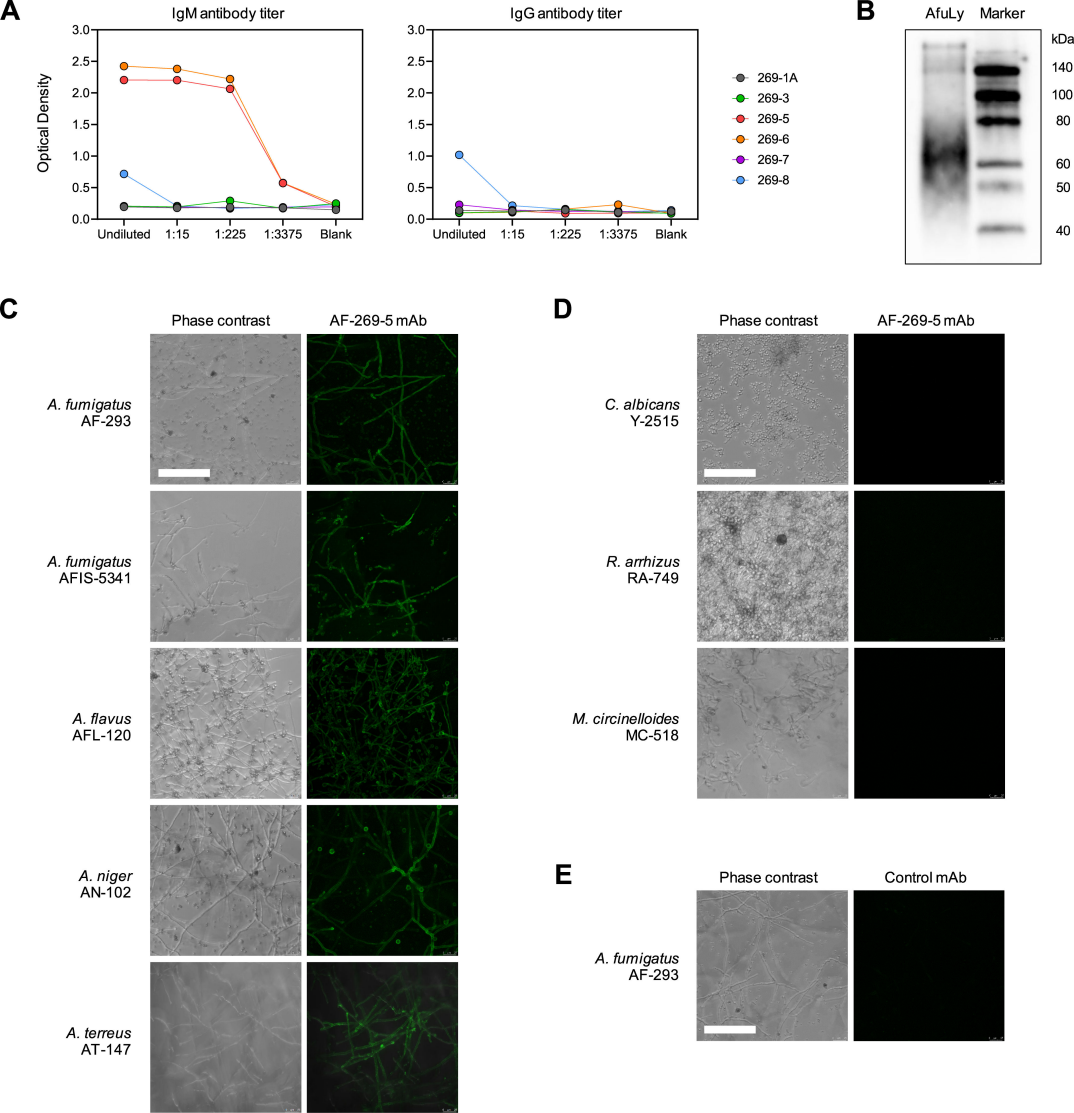
Screening and validation of a novel *Aspergillus*-targeting monoclonal antibody. (**A**) Hybridoma clones reacting to AF were selected by ELISA screening against a mycelial lysate of AF (AfuLy, Miltenyi Biotech, #170-076-131). Clones AF-269-5 and 269-6 showed similar IgM titers, and clone AF-269-5 was used for all further experiments. (**B**) Western blot analysis revealed binding of monoclonal antibody AF-269-5 to an approximately 60-kDa antigen of AF. (**C**) Fluorescence micrographs showing binding of monoclonal antibody AF-269-5 to several *Aspergillus* isolates, as indicated by green fluorescence (due to staining with a FITC-conjugated secondary antibody, see Supplementary Methods). (**D**) Fluorescence micrographs confirming absence of AF-269-5 binding to the commensal yeast *Candida albicans* and two representative Mucorales isolates. (**E**) Representative micrograph confirming the lack of fluorescence after incubation of AF-293 with hybridoma supernatant from a non-targeting clone and FITC-labeled secondary antibody. (**C–E**) Scale: 100 µM.

Firstly, we produced AF-targeting mAb clones by immunization of mice with an AF lysate (AfuLy) and generation of hybridomas, as detailed in Supplementary Methods. After thorough screening, we selected mAb AF-269-5, an IgM-isotype antibody (Fig. 1A) that recognizes an approximately 60-kDa antigen ubiquitously expressed on AF hyphae (Fig. 1B and C). MAb AF-269-5 showed high-affinity binding to hyphae of representative AF isolates as well as additional clinically relevant *Aspergillus species* (*A. terreus*, *A. niger*, and *A. flavus*), along with weak conidial binding (Fig. 1C). However, the antibody did not bind Mucorales isolates (*Rhizopus arrhizus* and *Mucor circinelloides*) and the commensal yeast *Candida albicans* (Fig. 1D). Using a non-targeting hybridoma supernatant as an additional control, we further confirmed that binding of AF-269-5 to *Aspergilli* was specific and fluorescent signals were not due to autofluorescence (exemplarily shown for AF-293 in Fig. 1E).

### Initial validation of our AF-CAR T-cell product

**Fig 2:**
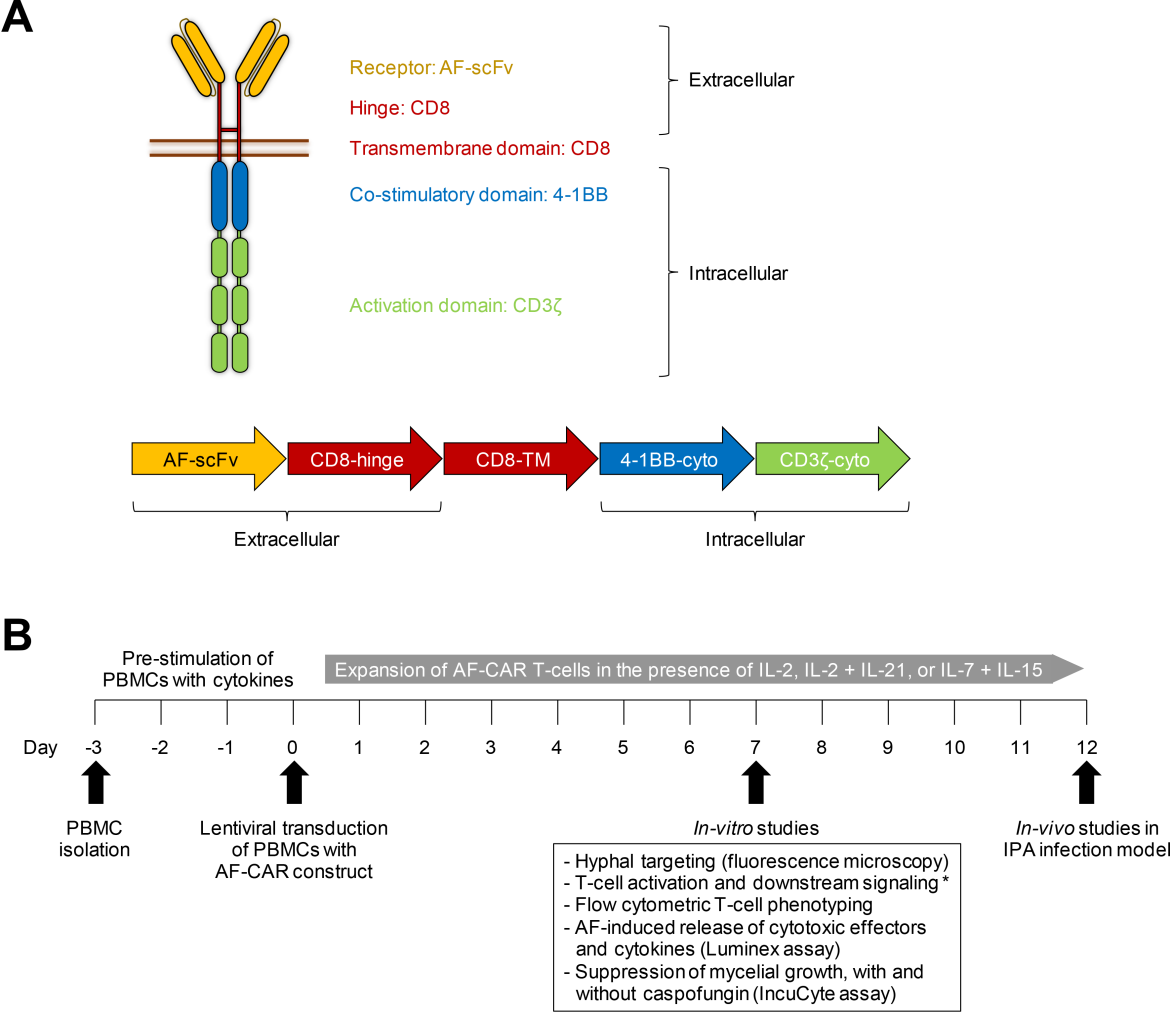
A novel lentiviral approach to generate AF-CAR T cells using the scFv of *Aspergillus fumigatus*-targeting monoclonal antibody AF-269-5. (**A**) Schematic of the AF-CAR construct. (**B**) Timeline for generation and testing of AF-CAR T cells. *T-cell activation and downstream signaling studies were performed in Jurkat *nuclear factor of activated T cells* (NFAT) reporter T cells, not in peripheral blood mononuclear cells (PBMCs). Abbreviations: AF, *Aspergillus fumigatus*; CAR, chimeric antigen receptor; CD, cluster of differentiation; cyto, cytoplasmatic domain; IL, interleukin; IPA, invasive pulmonary aspergillosis; NFAT, nuclear factor of activated T cells; PBMCs, peripheral blood mononuclear cells; scFv, single-chain variable fragment; TM, transmembrane domain.

Subsequently, we designed and developed a second-generation green fluorescence protein (GFP)-tagged AF-269-5-8a-CD137-CD3z CAR construct, whose extracellular part consisted of the scFv derived from mAb AF-269-5 (Fig. 2A), as detailed in Materials and Methods. All subsequent proof-of-concept experiments to characterize these cells and their antifungal activity focused on AF, the most common mold pathogen in immunosuppressed cancer patients (2).

**Fig 3:**
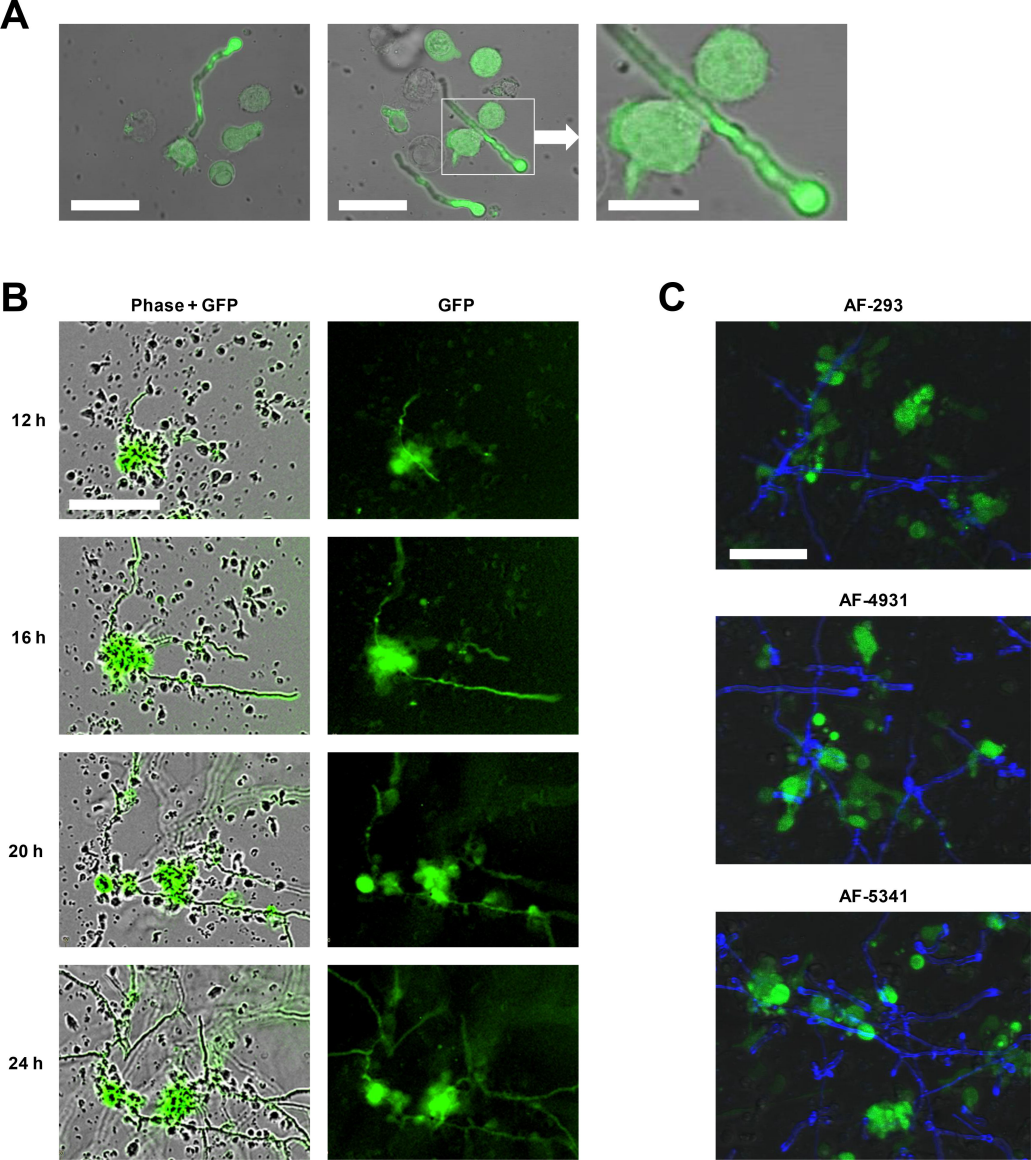
AF-CAR T cells target mature hyphae of *Aspergillus fumigatus*. (**A**) Fluorescence micrographs of GFP^bright^ AF-CAR T cells targeting the hyphal tip and cell wall of hyphal filaments of *A. fumigatus*. Scale: left and center image, 20 µm; right image, 10 µm. (**B**) Time-lapse micrographs showing robust early clustering of GFP^bright^ AF-CAR T cells at hyphal filaments of *A. fumigatus*, whereas GFP^dim^ cells target the hyphae more tardily or not at all. Scale: 100 µm. (**C**) Fluorescence micrographs of GFP^bright^ AF-CAR T cells targeting hyphal filaments of three different *A. fumigatus* isolates stained with Calcofluor (blue) after 4 hours of co-culture. Scale: 50 µm.

First, we sought to verify that our newly developed AF-CAR T cells can target mature AF hyphae. Therefore, primary human PBMCs were transduced with a “good manufacturing practice”-compliant LV expression system and co-cultured with AF-293-GFP. Fluorescence microscopy revealed that AF-CAR-expressing T cells (GFP^+^) protrude and interact with AF hyphae, whereas control T cells (not expressing GFP) did not interact with hyphal filaments (Fig. 3A). Formation of AF-CAR T-cell clusters in the vicinity of AF hyphae further corroborated successful targeting of mature hyphal filaments (Fig. 3B; Fig. S1; Movie S1). Targeting, as evidenced by the formation of cell clusters at hyphal filaments, was further confirmed with two additional azole-resistant AF isolates (Fig. 3C).

To validate AF-induced CAR signaling, the AF-CAR construct was transduced into Jurkat Lucia NFAT reporter cells. Approximately 92% of the transduced cells was GFP positive, indicating expression of the AF-CAR (Fig. S2A). Overnight stimulation with AfuLy concentration dependently induced luciferase production as a surrogate of CAR activation in AF-CAR-expressing cells, with means of 106.0 k RFUs (*P* = 0.006 versus unstimulated) and 147.7 k RFUs (*P* < 0.001 versus unstimulated) at 50 and 100 µg/mL AfuLy, respectively (Fig. S2B). In contrast, AfuLy stimulation did not significantly increase luciferase production by control Jurkat T cells (Fig. S2B). Similarly, only AF-CAR-expressing T cells but not control Jurkat T cells showed robust upregulation of the activation marker CD69 in response to AfuLy (Fig. S2C). Altogether, these pilot experiments establish successful targeting of AF germlings and mature hyphae and the induction of a functional downstream response upon stimulation with AF antigens.

### Phenotypes of AF-CAR T cells depending on the cytokine environment during expansion

Next, we sought to systematically assess the phenotypes and antifungal activity of AF-CAR T cells generated using protocols that mimic the production process for clinical cell therapies (24). To that end, AF-CAR T cells were generated using PBMCs from four healthy human donors. PBMCs isolated from buffy coats contained 66% ± 8% T cells (CD3^+^) and 10% ± 1% NK cells (CD3^−^CD56^+^), as determined by flow cytometry. Baseline CD4^+^ and CD8^+^ populations among viable CD3^+^ cells were 61% ± 7% and 29% ± 7%, respectively.

**Fig 4:**
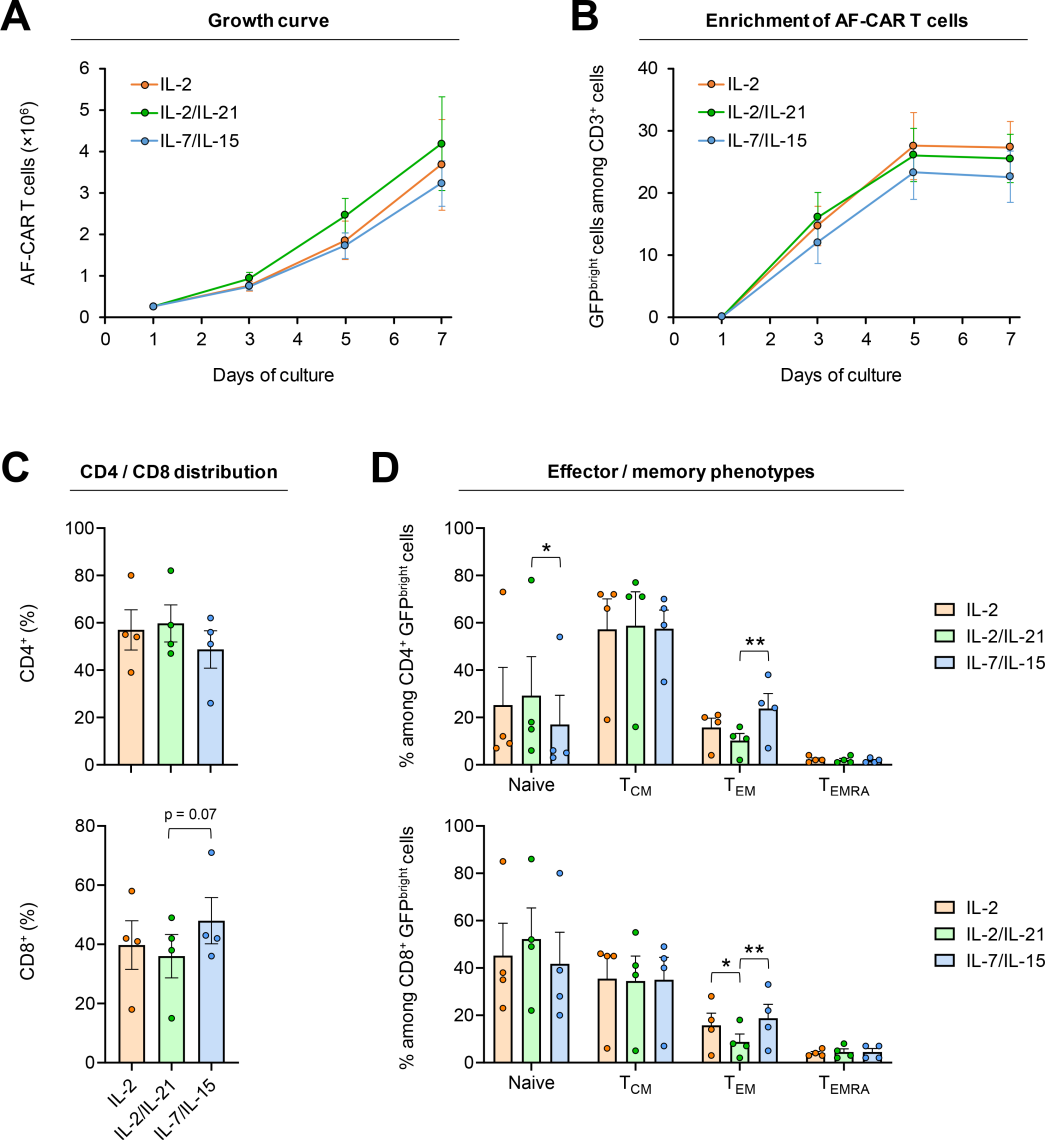
Three distinct cytokine regimens promote effective expansion of AF-CAR T cells and result in subtle differences in T-cellular phenotypes. (**A**) Absolute numbers of GFP^bright^ AF-CAR T cells after 3, 5, and 7 days of expansion, starting with 2.5 × 10^5^ transduced T cells. (**B**) Mean content of GFP^bright^ cells among all CD3^+^ T cells after 3, 5, and 7 days of expansion. (**C**) Content of CD4^+^ and CD8^+^ cells among GFP^bright^ CD3^+^ T cells after 7 days of expansion. (**D**) Effector/memory phenotypes among GFP^bright^ CD4^+^ T-helper cells and CD8^+^ cytotoxic T lymphocytes after 7 days of expansion. Naïve T cells, CCR7^+^ CD45RA^+^; central memory T cells (T_CM_), CCR7^+^ CD45RA^−^; effector memory T cells (T_EM_), CCR7^−^ CD45RA^−^; terminally differentiated effector memory T cells with re-expression of CD45RA (T_EMRA_), CCR7^−^ CD45RA^+^. (**A–D**) Columns and error bars denote mean and standard error of the mean, respectively. Repeated measures one-way analysis of variance with Tukey’s multiple comparison test. *P < 0.05 and **P < 0.01.

The *ex vivo* culture environment, especially the cytokine environment during expansion, critically affects differentiation, activity, and persistence of CAR T cells (25). We therefore expanded AF-CAR T cells for 7 days in the presence of either IL-2 alone, IL-2 + IL-21, or IL-7 + IL-15. We found 13- to 17-fold expansion of AF-CAR T cells with either of the three cytokine regimens, with the IL-2-containing regimens yielding a slightly higher number of viable AF-CAR T cells (Fig. 4A). Similarly, enrichment of AF CAR-expressing cells was marginally higher with the IL-2-containing regimens, with an average of 26%–27% versus 23% (IL-7 + IL-15) GFP^bright^ (i.e., strongly CAR-expressing) cells among CD3^+^ cells (Fig. 4B). However, both observations did not reach statistical significance.

CD3^+^ T cells consistently accounted for >95% of the cell population post-transduction and during *in vitro* expansion. The IL-7/IL-15 regimen yielded a higher proportion of cytotoxic T cells (CD8^+^ cells) among CD3^+^ T cells (48% versus 36%–40%), although significance was not reached for this observation (Fig. 4C). We further observed a higher percentage of effector memory AF-CAR T cells and a lower proportion of naïve CD4^+^ cells after IL-7 + IL-15 stimulation compared with IL-2- or IL-2 +IL-21-expanded cells (Fig. 4D).

**Fig 5:**
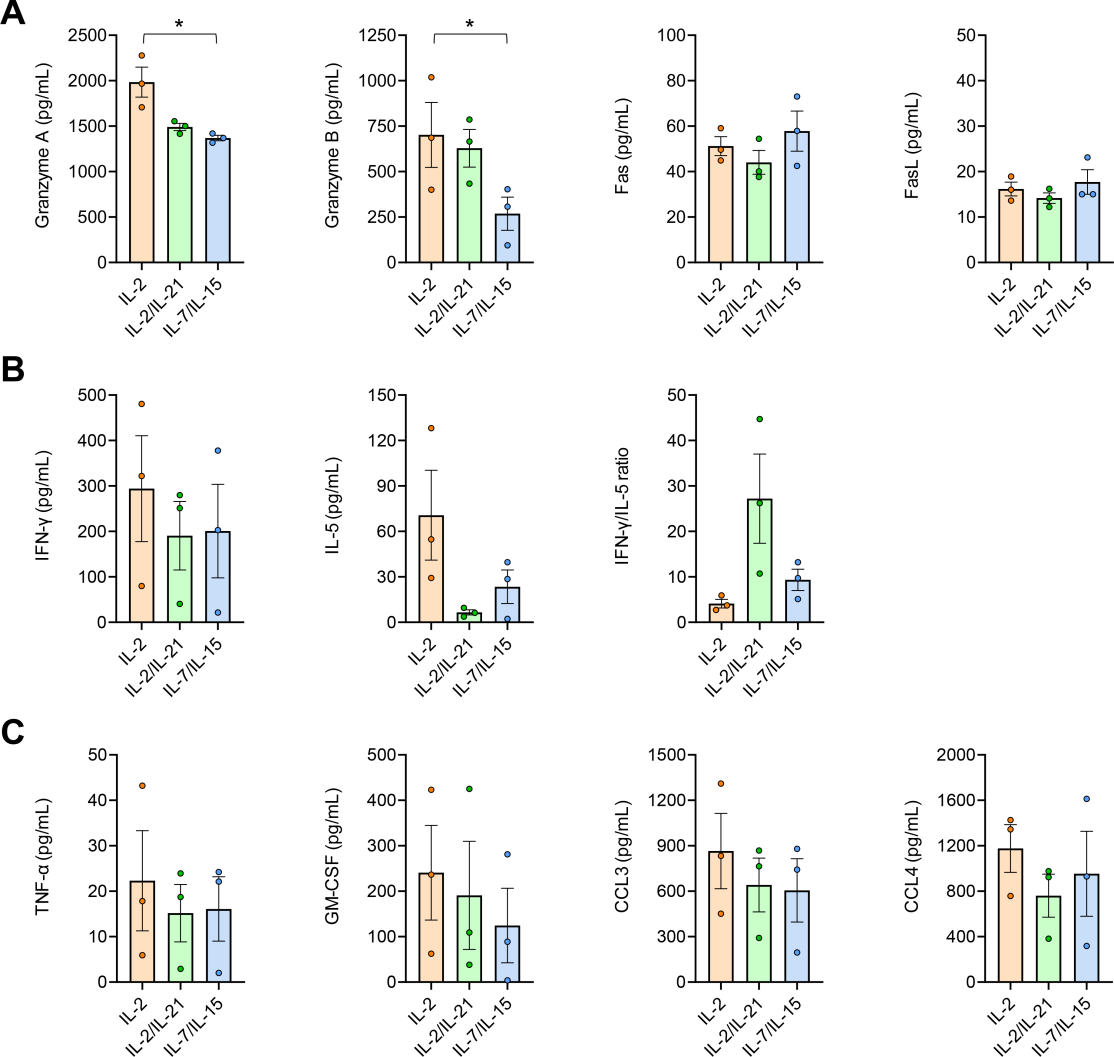
AF-CAR T cells show potent release of cytotoxic effectors and immunostimulatory cytokines upon co-culture with *Aspergillus fumigatus in vitro*. Concentrations of cytotoxic effectors (**A**), type 1 and type 2 T-cell signature cytokines (**B**), and immunostimulatory cytokines and chemokines (**C**) in supernatants of 24-hour co-cultures of 200,000 AF-CAR T cells with 200,000 *A*. *fumigatus* AF-293 conidia were determined with a 16-plex Luminex assay. *N* = 3 independent donors. Columns and error bars represent mean and standard error of the mean, respectively. Repeated-measures one-way analysis of variance with Tukey’s post-test. *P < 0.05. Secretion of IL-4, IL-6, IL-10, and IL-13 was below the detection threshold. IL-2 has been excluded from the analysis due to potential bias caused by remainders of recombinant IL-2 in IL-2-expanded cell preparations. 4-1BB concentrations were <20 pg/mL for all donors and expansion regimens (not displayed).

Furthermore, we performed a Luminex assay to determine the impact of the three AF-CAR T-cell expansion regimens on AF-induced release of cytokines and cytotoxic effectors (Fig. 5). Expansion of cells with IL-2 alone resulted in the strongest production of cytotoxic effectors granzyme A and granzyme B, especially when compared with the IL-7 + IL-15 regimen (*P* = 0.030 for granzyme A and *P* = 0.042 for granzyme B; Fig. 5A). In contrast, concentrations of Fas and FasL were largely comparable between the three expansion regimens. Although statistical significance was not reached, the IL-2 expansion regimen favored the release of IFN-γ (Fig. 5B), a cytokine associated with favorable polarization of anti-AF immunity (6), as well as other effector cytokines and chemokines linked to innate immune cell recruitment (i.e., CCL3, CCL4, and GM-CSF; Fig. 5C). AF-CAR T-cell expansion in the presence of IL-2 further elicited the strongest IL-5 response, albeit at a >fourfold lower concentration than IFN-γ (Fig. 5B). Except for IL-5, there was no detectable secretion of cytokines associated with type 2 T-helper cell (IL-4, IL-13) or regulatory T-cell responses (IL-10), indicating an overall favorable, type-1-prone (Th1/Tc1) polarization of the cytokine response. The highest IFN-γ/IL-5 gradient (mean, 27.1) was found after IL-2 + IL-21 stimulation (Fig. 5B).

Taken together, all three expansion regimens yielded mostly comparable phenotypes. AF-CAR T cells expanded with IL-7 + IL15 showed increased frequencies of more differentiated memory subsets, whereas the IL-2-containing regimens produced slightly higher AF-CAR T-cell yields and stronger release of cytokines and cytotoxic effectors in response to AF stimulation.

### Antifungal activity of AF-CAR T cells *in vitro*

**Fig 6:**
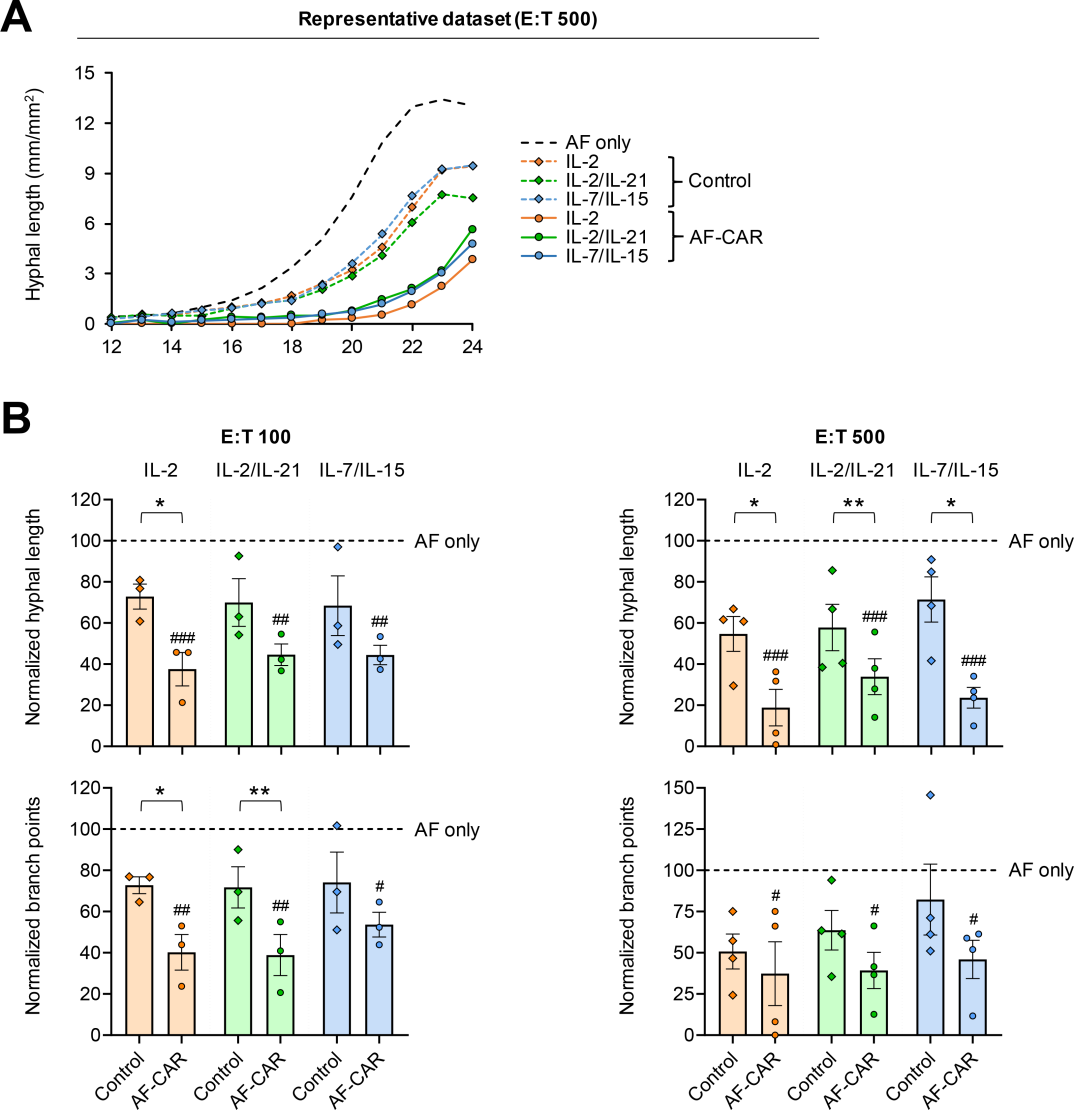
AF-CAR T cells suppress the growth of *Aspergillus fumigatus in vitro*. AF-CAR T cells from four independent donors were expanded for 7 days in media supplemented with three different cytokine cocktails. CAR T cells were co-cultured with GFP-expressing AF conidia at different effector-to-target (E:T) ratios. Mycelial expansion was tracked using the IncuCyte NeuroTrack module. Hyphal length per mm^2^ in co-cultures was normalized to the average hyphal length in “AF only.” (**A**) A data set from one representative donor is shown to illustrate the impact of control T cells and AF-CAR T cells on mycelial expansion over time, depending on the cytokine regimen used for AF-CAR T-cell expansion. (**B**) Hyphal length and branch point numbers were normalized to an “AF only” control without T cells. *N* = 3 (E:T 100) or 4 (E:T 500) independent donors. Normalized individual results, mean (column height), and standard error of the mean (error bars) are shown. Repeated-measures one-way analysis of variance with Dunnett’s multiple comparison test was used to compare AF-CAR T-cell co-cultures with the “AF only” control. #*P* < 0.05, ##*P* < 0.01, and ###*P* < 0.001. In addition, paired two-sided Student’s *t*-test was used to compare control and AF-CAR T cells expanded with the same cytokine cocktail. *P < 0.05 and **P < 0.01. Of note, the IncuCyte NeuroTrack methodology requires flat-bottom plates for precise focusing and tracking; hence, E:T ratios needed for significant fungal inhibition are inflated at least by factor 5–10 compared with conventional (e.g., metabolic) assays commonly performed in round- or V-bottom plates.

To compare the antifungal activity of AF-CAR T cells depending on the expansion regimen, we performed time-lapse imaging with automated analysis of hyphal growth and morphogenesis (26). In the absence of immune cells, AF-293 reached its maximum mycelial density at the bottom of the wells, where the measurement was taken, after 22–24 hours (representative example shown in Fig. 6A). Control T cells did not delay the growth plateau but reduced the maximum mycelial density, presumably due to competition for space and nutrients. In contrast, no growth plateau was reached after 24 hours in the presence of AF-CAR T cells and mycelial expansion was further suppressed compared with co-cultures with control T cells from the same donor (Fig. 6A).

Compared to AF alone, a 100-fold E:T ratio of AF-CAR T cells suppressed mycelial length and branching after 18 hours by 55%–62% and 46%–61%, respectively (*P* < 0.05 for all three cytokine regimens and both readouts; Fig. 6B). The incremental inhibition of mycelial proliferation compared with control T cells was 24 to 35 percentage points for hyphal length (*P* = 0.005 for IL-2) and 20 to 33 percentage points for mycelial branching (*P* = 0.022 for IL-2 and *P* = 0.004 for IL-2/IL-21; Fig. 6B). At a 500-fold E:T ratio, suppression of mycelial proliferation by AF-CAR T cells was at 66%–81% for hyphal length (*P* < 0.001 for all three cytokine regimens) and at 54%–63% for hyphal branching (*P* < 0.05 for all three cytokine regimens; Fig. 6B). Compared with an equal number of control T cells, mean incremental inhibition of hyphal length after 18 hours of co-culture was 36 percentage points for IL-2 (*P* = 0.022), 24 percentage points for IL-2/IL-21 (*P* = 0.009), and 48 percentage points for IL-7/IL-15 (*P* = 0.012; Fig. 6B). IL-7/IL-15 also produced the strongest incremental effect for suppression of mycelial branching (36 percentage points) by AF-CAR T cells versus control T cells, although significance was not reached (Fig. 6B).

**Fig 7:**
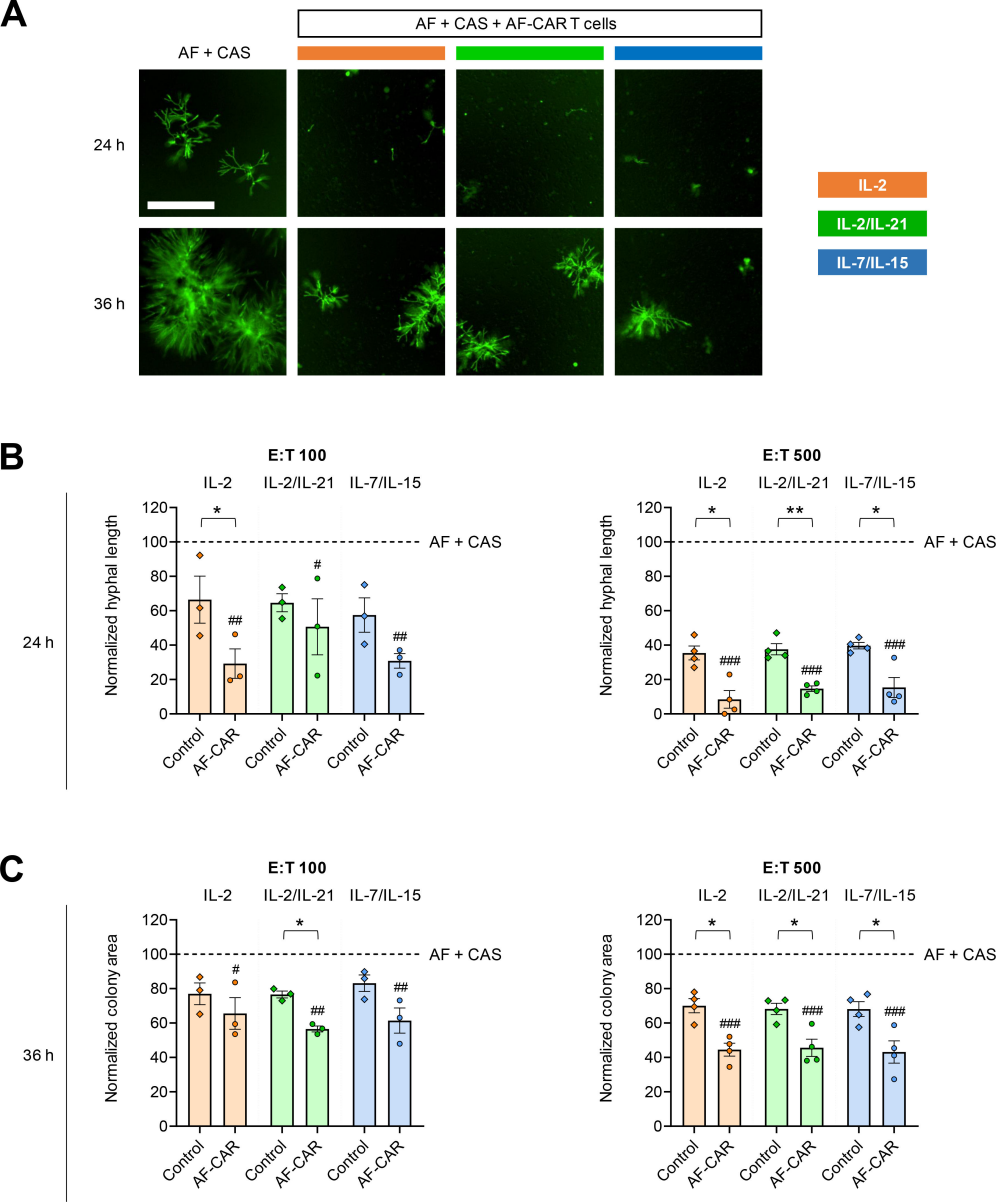
AF-CAR T cells synergize with the immunomodulatory antifungal agent caspofungin to elicit sustained suppression of *Aspergillus fumigatus* growth *in vitro*. AF-CAR T cells from four independent donors were expanded for 7 days in media supplemented with three different cytokine cocktails. CAR T cells were co-cultured with GFP-expressing AF conidia at different E:T ratios in the presence of CAS at its minimum effective concentration against AF-293 (0.25 µg/mL). Mycelial expansion was tracked using the IncuCyte NeuroTrack module. Additionally, diameters of individual AF-293 colonies in CAS-supplemented co-cultures were determined using the manual measurement tool of the IncuCyte ZOOM software. Colony area was approximated using the following formula: colony area = π × (0.5 × diameter)^2^. At least 20 representative colonies were measured per condition. (**A**) Representative images of *A. fumigatus* colonies displaying the typical CAS-induced rosette morphology. Reduction in colony size compared with *A. fumigatus* + CAS alone indicates an inhibitory effect of AF-CAR T cells on fungal proliferation. (**B and C**) Hyphal length after 24 hours (**B**) and mean colony area after 36 hours (**C**) were normalized to an “AF + CAS” control. *N* = 3 (E:T 100) or 4 (E:T 500) independent donors. Normalized individual results, mean (column height), and standard error of the mean (error bars) are shown. Repeated-measures one-way analysis of variance with Dunnett’s multiple comparison test was used to compare AF-CAR T-cell co-cultures with the “AF + CAS” control. #*P* < 0.05, ##*P* < 0.01, and ###*P* < 0.001. Paired two-sided Student’s *t*-test was used to compare control and AF-CAR T cells expanded with the same cytokine cocktail. *P < 0.05 and **P < 0.01.

Additionally, we performed co-culture experiments in the presence of caspofungin (CAS) to facilitate tracking of fungal proliferation for extended periods and to test the activity of AF-CAR T cells when added to an immunomodulatory antifungal previously shown to synergize with anti-AF immunotherapy (27). We utilized the minimum effective concentration (MEC) of CAS against the tested isolate, i.e., a drug concentration eliciting the characteristic change to a rosette-like colony morphology (26) but not entirely inhibiting mycelial proliferation (Fig. 7A). Importantly, binding of the AF-269-5 mAb to AF hyphae was not abrogated or impaired by CAS pretreatment (Fig. S3).

Normalized to AF cultured in the presence of CAS but without immune cells, an E:T 100 ratio of AF-CAR T cells suppressed mycelial length after 24 hours by 71% (IL-2), 49% (IL-2/IL-21), and 69% (IL-7/IL-15). As in the experiment without CAS (Fig. 6B), the strongest incremental antifungal activity compared with control T cells was elicited by IL-2-expanded AF-CAR T cells (37 percentage points, *P* = 0.024; Fig. 7B). At an E:T ratio of 500, AF-CAR T cells expanded with either cytokine regimen suppressed mycelial proliferation by at least 85% compared with CAS alone (*P* < 0.001 for all three cytokine conditions). Incremental activity compared with control T cells was largely comparable among the three cytokine regimens and was 23–27 percentage points (*P* = 0.003–0.037; Fig. 7B).

After 36 hours of co-culture, when the hyphal filaments within the colonies became too dense for NeuroTrack analysis, we used the colony area as a surrogate of mycelial proliferation (Fig. 7C). AF-CAR T cells reduced the colony area by 54%–57% at an E:T ratio of 500 (*P* < 0.001 versus CAS alone for all three cytokine regimens; Fig. 7C). With either cytokine expansion regimen, the incremental antifungal activity of AF-CAR T cells (versus control T cells) against CAS-exposed AF was 23–26 percentage points after 36 hours of co-culture at E:T 500 (*P* = 0.028–0.031; Fig. 7C). Altogether, these findings suggest sustained antifungal *in vitro* activity of AF-CAR T cells expanded using either of the three cytokine regimens, especially when combined with CAS.

### Evaluation of therapeutic *in vivo* activity of AF-CAR T cells in a murine model of IPA

**Fig 8:**
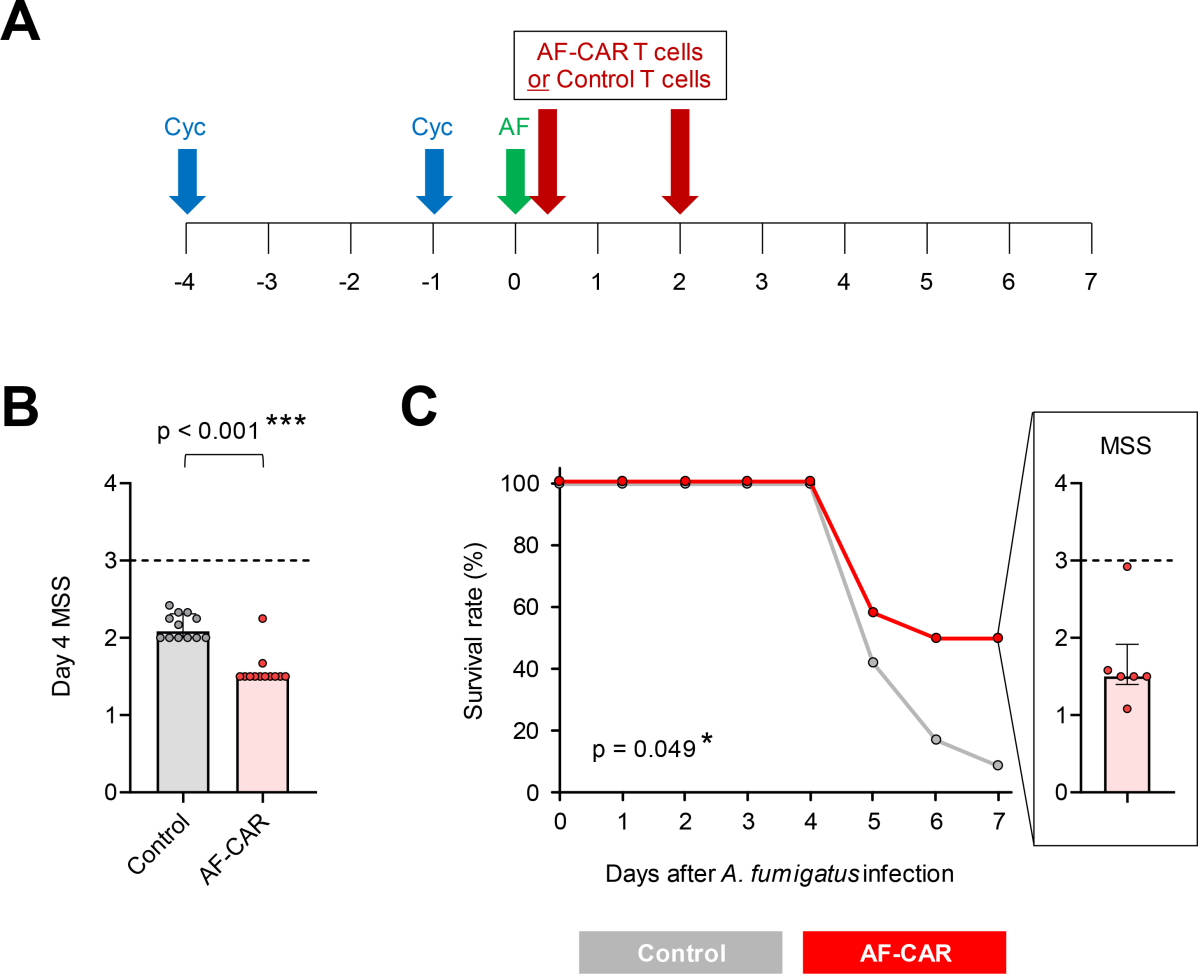
Immunotherapy with AF-CAR T cells provides a significant therapeutic benefit in mice with invasive pulmonary aspergillosis. (**A**) Timeline of experimental interventions. Eight-week-old female NSG received two doses of cyclophosphamide (Cyc) to induce neutropenia and were infected intranasally with *A. fumigatus* AF-293 conidia (AF), as described in Materials and Methods. On the day of infection (6 hours post-infection) and on day +2, mice received intravenous injections of 5 × 10^6^ AF-CAR T cells or control T cells from the same donor. Survival was monitored for 7 days post-infection. Infection severity was scored on days +4 and +7 using the modified murine sepsis score (MSS). Aggregate results from two replicate experiments with a total of *N* = 12 mice per treatment are shown. (**B**) MSS distributions (median and inter-quartile range) of infected mice on day +4 depending on the treatment received. Mann-Whitney U test. (**C**) Survival curves of infected mice depending on the treatment received. Mantel-Cox log-rank test. The insert shows MSS scores of the six survivors in the AF-CAR T-cell therapy cohort on day +7.

Lastly, we performed a pilot *in vivo* study to test the tolerability and therapeutic efficacy of AF-CAR T cells in NOD.Cg-Prkdc^scid^ Il2rg^tm1Wjl^/SzJ (NSG) mice with IPA. NSG mice have defective T- and B-cell immunity, allowing for xenogeneic cell transfer, and were additionally immunosuppressed with cyclophosphamide to mimic chemotherapy-induced neutropenia (Fig. 8A). To generate AF-CAR T cells for *in vivo* therapy, we opted for a sequential cytokine expansion regimen of culturing CAR T cells with IL-2 for 6 days, followed by stimulation with CD2, CD3, and CD28 antibodies in media supplemented with IL-2, IL-7, and IL-15 for another 6 days.

Expectedly, mice with IPA receiving non-targeting control T cells developed considerable distress by day 4 post-infection (MSS of 2.1; Fig. 8B) and 11 out of 12 mice receiving mock treatment with control T cells died by day 7 post-infection (Fig. 8C). AF-CAR T-cell infusions were well tolerated, with no noticeable signs of distress after intravenous injection. Treatment with AF-CAR T cells reduced the median day 4 MSS of infected mice to 1.5 (*P* < 0.001 versus mock treatment; Fig. 8B), indicating a significant and consistent early therapeutic benefit. Moreover, AF-CAR T-cell therapy conferred a significant survival benefit through day +7 compared with mock treatment (survival rate, 50% vs. 8%, *P* = 0.049; Fig. 8C). Of note, five out of six survivors in the AF-CAR T-cell therapy cohort had stable infection severity compared with day +4 (median MSS, 1.5; Fig. 8C). Taken together, AF-CAR T-cell therapy was well tolerated and conferred significant short-term protection in severely immunocompromised mice with IPA, even in the absence of concomitant antifungal therapy.

## DISCUSSION

New immunotherapies are direly needed to counteract immune paralysis in immunocompromised patients with IA. Antifungal CAR T cells were proposed as a promising approach, because they have the potential to both directly kill the invading fungus and galvanize the host’s innate immune system into action. Herein, we developed a new strategy to design improved AF-CAR T cells using the scFv domain of the AF-targeting mAb AF-269-5 and an LV expression system.

This newly developed construct has several major advantages in its design and mode of action compared with published AF-reactive CAR T-cell products (14–16): (i) unlike Dectin-1 CAR T cells that have weak affinity to mature mycelium, our present product was designed to efficiently target mature AF hyphae; (ii) the spacer domain of the newly developed AF-CAR has been derived from the alpha chain of CD8, which was shown to increase CAR persistence in the circulation (28). In contrast, the hIgGFc spacer domain used in previously generated antifungal CAR T-cells (14) might be more susceptible to clearance by Fc receptors (29); (iii) the CD137/4-1BB costimulatory domain was selected based on its potential clinical benefits of improved persistence, enhanced proliferation, and lower propensity to induce immunotoxicity compared with the previously used costimulatory molecule CD28 (30, 31); and (iv) our AF-CAR T cells were generated using an LV vector system that could accelerate the generation of clinical-grade AF-CAR T cells.

Hyphae are the predominant morphotype in AF-infected tissue (2). Therefore, we verified hyphal targeting by microscopic analyses confirming binding and formation of AF-CAR T-cell clusters at AF hyphae, assessment of downstream NFAT signaling after stimulation with a hyphal lysate, and measurement of functional cytokine responses in the culture supernatant after co-culture with preformed AF hyphae.

AF-CAR T cells expanded with either of the three studied cytokine regimens elicited potent anti-AF effector cytokine responses. Although gradual differences were seen depending on the expansion regimen, especially stronger release of cytotoxic effectors and key cytokines for innate immune cell recruitment after IL-2-supplemented expansion, we found an overall favorable, type 1-prone polarization of the cytokine milieu produced by the AF-CAR T cells with either of the three expansion regimens. Unlike another recently published AF-CAR T-cell product (16), our cells showed minimal release of type-2 T-cell cytokines and no detectable secretion of IL-10, the signature cytokine of regulatory T cells. However, the concordance of T-cell polarization *in vitro* and the therapeutic efficacy of anti-infective CAR T-cell treatments remain to be established (32, 33).

The observation of enhanced and more sustained antifungal *in vitro* activity of AF-CAR T cells in the presence of effective CAS concentrations was likely due to significantly delayed mycelial expansion, providing a longer time frame for the AF-CAR T cells to mount a potent effector response against the fungus. In addition, true immuno-synergistic effects between CAS and AF-CAR T cells would be conceivable. Specifically, glucan depletion of the AF cell wall after CAS exposure could enhance the accessibility of the thus far uncharacterized target antigen while also contributing to the co-activation of innate effector cells through exposure of branched β-glucans (34).

Based on our cumulative *in vitro* findings, we selected a sequential cytokine stimulation regimen with IL-2, followed by a combination of IL-2, IL-7, and IL-15, to generate AF-CAR T cells for therapeutic *in vivo* studies. To mimic a state of severe and pleiotropic immunosuppression frequently seen in high-risk patients with HM, these experiments were conducted in mice developing IPA in a setting of adaptive immune dysfunction combined with chemotherapy-induced neutropenia and in the absence of antifungal pharmacotherapy. Unlike a previous study (16), we used cyclophosphamide instead of a neutrophil-depleting antibody to introduce neutropenia in NSG mice, in order to assess the efficacy of AF-CAR T cells in the presence of a cytotoxic agent that might hamper T-cell viability and functionality. Encouragingly, our CAR T-cell product was well tolerated, and our data revealed a significant morbidity/mortality benefit of these cells, even in a severely immunocompromised host environment with the presence of cytotoxic chemotherapy and without concomitant antifungal chemotherapy.

This proof-of-concept study has limitations. Firstly, the specific target antigen and the many conceivable strain-, morphotype-, host-related, and iatrogenic factors (e.g., antifungal therapy) that could affect its expression remain unknown. The lack of a distinct band in our immunoblot (Fig. 1B) and the observed cell wall labeling suggest that the target might contain a glycan epitope (e.g., a glycoprotein), but further studies will be needed for detailed antigen characterization. Secondly, while the AF-269-5 mAb bound to representative non-AF *Aspergilli*, this study solely focused on AF and did not characterize the *in vitro* or *in vivo* efficacy of the actual AF-CAR T cells against additional *Aspergillus* species. Although AF is the predominant cause of IA, a pan-*Aspergillus* or even more broadly targeting CAR T-cell product could greatly expand the clinical utility of AF-CAR T cells. Thirdly, although a unique strength of our study was the assessment of *in vitro* activity of AF-CAR T cells in the presence of CAS, we did not study interactions of AF-CAR T cells and AF with concomitant exposure to first-line antifungals (i.e., voriconazole) and did not test the therapeutic benefit of AF-CAR T cells in the setting of antifungal chemotherapy *in vivo*. Furthermore, our pilot *in vivo* study in a limited number of mice did not include testing of the *in vivo* persistence of the infused CAR T cells, *in vivo* imaging to confirm lung tissue targeting, or detailed assessment of off-target toxicities. Additional limitations of our mouse model include the relatively short follow-up period and the administration of the first therapeutic infusion only 6 hours after infection, before a full-blown infection had been established. Lastly, although demonstrating potent immunostimulatory cytokine responses of AF-CAR T cells *in vitro*, we did not assess the immunostimulatory effect of AF-CAR T cells on allogenic innate effector cells *in vitro* or *in vivo*. Notably, conclusive studies of the interplay between AF-CAR T cells and natural host immunity are difficult to perform in NSG mice due to their pleiotropic immune defects and the xenogeneic nature of the AF-CAR T-cell product. However, testing in less artificial mouse models would have required expensive and challenging production of murine CAR T cells, which was beyond the scope of this pilot study.

In conclusion, we have developed a novel lentiviral approach to generate AF-CAR T cells targeting mature AF hyphae. The resulting AF-CAR T cells showed high targeting efficacy and yielded significant short-term protection in mice with IPA. As with other strategies to generate antifungal CAR T cells previously proposed by us and others, many unknowns remain to be addressed to possibly facilitate future clinical translation. For instance, critical gaps of knowledge include optimal dosing of CAR T cells, optimal sequencing with first-line antifungal chemotherapy, and potential synergies with other antifungal immunotherapies, such as cytokines or immune checkpoint inhibitors (35). Furthermore, to inform optimal patient selection, future *in vivo* studies will need to characterize synergies of AF-CAR T cells with the autochthonous host response depending on the type and severity of underlying immune dysfunction. Because the need for cell expansion limits the use as a “real-time” intervention against an aggressive opportunistic infection, development of allogeneic antifungal CAR T cells for immediate use as an “off-the-shelve” therapy would be warranted. Lastly, our approach might open new avenues to design AF-CAR NK cells or AF-CAR macrophages to overcome common limitations of CAR T cells such as their propensity to induce CRS.

## MATERIALS AND METHODS

### Fungal culture

Fungal strains used for this study and their sources are summarized in Table S1. Strains were cultured on potato dextrose agar plates for 3–5 days at 37°C. Conidia/spores were harvested by overlaying the mycelium with sterile phosphate-buffered saline (PBS) and scraping with a sterile cell spreader. Conidia/spore suspensions were passed through a 40-µm cell strainer (Corning Falcon, Corning, USA) to remove residual mycelium. Conidia/spores were washed with sterile PBS and quantified with a hemocytometer.

### Generation of mAb AF-269-5

The AF-targeting mAb AF-269-5 was generated and characterized as detailed in Supplementary Methods and patent application WO2020163695A1.

### AF-CAR construct

The DNA sequence encoding the light and heavy chains of antibody AF-269-5 mAb was used to design the extracellular targeting domain of the second-generation AF-CAR construct. The targeting domain was fused to the hinge and transmembrane domains of human CD8α and the cytoplasmic signaling domains of CD137/4-1BB and CD3ζ (36). To monitor CAR expression, a GFP sequence was added at the C-terminus of the AF-CAR construct with self-splicing spacer 2A (37). The full-length AF-CAR construct containing GFP was subcloned into the third-generation self-inactivating LV vector dCAS9-VP64-GFP (Addgene, Water Town, USA) under the control of the EF1α promoter (Fig. S4), as previously described (15). The presence of the AF-CAR construct was verified by double digestion with NheI and BSIWi. Endotoxin-free AF-CAR pDNA was prepared using the Plasmid Maxi Kit (Qiagen, Hilden, Germany) following the manufacturer’s instructions.

### Production of AF-CAR LV particles

HEK293T cells (#ACC 635; DSMZ, Brunswick, Germany) were cultured in Dulbecco’s modified Eagle’s medium (DMEM, Biochrom, Berlin, Germany) supplemented with 10% heat-inactivated fetal bovine serum (FBS), 100 U/mL penicillin, and 100 µg/mL streptomycin. AF-CAR containing LV particles were generated by transfection of 1 × 10^7^ HEK293T cells with AF-CAR pDNA using the Lipofectamine 3000 reagent (Thermo Fisher Scientific, Waltham, USA). The viral supernatant of LV-CAR-transfected HEK293T cells was collected daily for 3 days. The pool of viral particles was concentrated using the Lenti-X concentrator (Takara Bio USA Inc., Mountain View, USA). The transduction efficiency and AF-CAR viral titers were estimated by serial dilution transduction of AF-CAR lentiviral particles with HEK293T cells as described before (15). AF-CAR LV particles were aliquoted and cryopreserved at −80°C.

### Production of human AF-CAR T cells

Buffy coats were provided by the MD Anderson Cancer Center Blood Bank (Houston, USA) and were obtained from whole blood donations of healthy adult donors. PBMCs were isolated using Ficoll-Paque PLUS solution (Cytiva, Marlborough, USA) according to the manufacturer’s instructions. PBMCs were stimulated with an antibody cocktail (ImmunoCult human CD3/CD28 T-cell activator; STEMCELL Technologies, Cambridge, USA) in the presence of IL-2 (Peprotech, Cranbury, USA, 250 IU/mL), IL-2 (150 IU/mL) plus IL-21 (Peprotech, 15 ng/mL), or IL-7 (Peprotech, 5 ng/mL) plus IL-15 (Peprotech, 5 ng/mL) for 3 days. For transduction, 12-well tissue culture plates were coated with Retronectin (Takara Bio USA, Sam Jose, USA; 10 µL of 1 µg/mL Retronectin in PBS per well) overnight, loaded with 100 µL of concentrated LV particles, and centrifuged for 90 min at 1,500 × *g*, 32°C. Thereafter, 2.5 × 10^5^ activated PBMCs were added to each well. Plates were centrifuged for 45 min at 800 × *g*, 32°C. On the following day, cells were washed with fresh Roswell Park Memorial Institute (RPMI) medium and incubated with the respective cytokine cocktails for up to 12 days. Transduction efficiency was analyzed by flow cytometry on days 3, 5, and 7 post-transduction.

### Fluorescence microscopy

AF-CAR T cells and control T cells were co-cultured with AF germlings at an E:T ratio of 100:1. After 6 hours of co-culture at 37°C, interactions were visualized using a confocal fluorescent microscope (SP8, Leica Biosystems, Wetzlar, Germany) at 10× and 63× magnification. For some imaging studies, hyphae were stained with Calcofluor (Sigma-Aldrich, St. Louis, USA). Fluorescence microscopy studies of AF-269-5 antibody binding to fungal cells are described in Supplementary Methods.

### Flow cytometry

Cells were stained with Aqua Live/Dead viability dye (Life Technologies, Carlsbad, USA), followed by extracellular antibody staining for 30 min at room temperature. Antibodies, clones, fluorochromes, and manufacturers are summarized in Table S2. Samples were analyzed with a BD LSR Fortessa flow cytometer using BD FACSDiva software (BD Bioscience, Franklin Lakes, USA). Data were analyzed using FlowJo v10.2 (FlowJo LLC, Ashland, USA). The gating strategy for phenotypic analyses of AF-CAR T cells and a representative data set are shown in Fig. S5.

### Analysis of AF-induced cytokine release

AF-CAR T cells and control T cells were cultured overnight in serum- and cytokine-free medium. On the following day, 200,000 cells were co-cultured with the same number of AF-293 conidia in 400 µL RPMI + 10% FBS for 24 hours at 37°C. Supernatant was collected, and cytokine concentrations were measured using the Human Luminex Discovery Assay 16-plex Kit (LXSAHM-16, Bio-Techne Corporation, Minneapolis, USA) according to the manufacturer’s protocol. Plates were analyzed with a Luminex 200 device (Luminex Corporation, Austin, USA).

### IncuCyte assay

AF-293-GFP conidia and AF-CAR T cells or control T cells were co-cultured at E:T ratios of 1:100 and 1:500 in a 96-well flat bottom plate (Greiner Bio-One, Kremsmünster, Austria) containing growth media (RPMI + 10% FBS). For synergistic studies, 0.5 µg/mL CAS (Sigma-Aldrich, St. Louis, USA; final concentration = 0.25 µg/mL, equaling the MEC of CAS against AF-293) or drug-free RPMI + 10% FBS was added. Each co-culture condition was tested in duplicate. Controls included (i) conidia without T cells, with or without CAS (to determine baseline fungal growth for normalization), (ii) T cells without conidia (to subtract background fluorescence caused by GFP in the AF-CAR construct), and (iii) cell-free medium (sterility control). Plates were imaged hourly for 36 hours at 37°C using the IncuCyte ZOOM time lapse microscopy system (Sartorius, Göttingen, Germany). Mycelial expansion was quantified using the “Green Channel Neurite Length” NeuroTrack feature, as previously described (26).

### *In vivo* studies

Eight-week-old female NSG mice (Charles River Laboratories, Wilmington, USA) weighing 18–20 g received intraperitoneal injections of cyclophosphamide (150 mg/kg on day −4 and 100 mg/kg on day −1) to induce neutropenia. Mice were then infected intranasally with 1.5 × 10^6^ AF-293 conidia on day 0. On the day of infection (6 hours post-infection) and on day +2, mice received intravenous injections (tail vein) of 5 × 10^6^ AF-CAR T cells or control T cells from the same donor, formulated in a volume of 200 µL sterile saline. Survival was monitored for 7 days post-infection. Infection severity was scored using the modified murine sepsis score, as previously described (38, 39).

### Statistical analyses

At least 3–4 technical or biological replicates (as appropriate) were performed for all *in vitro* experiments. Statistical analyses were performed using Microsoft Excel and GraphPad Prism version 9.0 (GraphPad software, San Diego, USA). Significance testing was performed using unmatched or repeated measures one-way analysis of variance with Tukey’s multiple comparison test or unpaired two-tailed *t*-test, as detailed in the figure legends. Survival curves were compared using the Mantel-Cox log-rank test.

## SUPPLEMENTAL MATERIAL

Supplemental Material mbio.03413-23-s0001.docx Supplemental text, figures, movie legend, and tables.

Movie S1 mbio.03413-23-s0002.mp4 AF-CAR T cells target A. fumigatus hyphae and form clusters at mature mycelium.

ASM does not own the copyrights to Supplemental Material that may be linked to, or accessed through, an article. The authors have granted ASM a non-exclusive, world-wide license to publish the Supplemental Material files. Please contact the corresponding author directly for reuse.
