# Supplementary material for: A novel lentiviral vector-based approach to generate chimeric antigen receptor T cells targeting Aspergillus fumigatus
Source: mBio. 2024 Feb 28;15(4):e03413-23. doi: 10.1128/mbio.03413-23 (PMC11005356; doi:10.1128/mbio.03413-23)
Supplement: Supplemental Material — Supplemental text, figures, movie legend, and tables. [file mbio.03413-23-s0001.docx]

A novel lentiviral vector-based approach to generate chimeric antigen receptor T cells targeting *Aspergillus fumigatus*

**Supplementary Materials**

**Supplementary Methods**

1. **Generation and validation of monoclonal *Aspergillus* *fumigatus* antibody AF-269-5**

Immunization of mice and generation of hybridomas

Immunization and hybridoma generation were conducted at the University of Texas MD Anderson Cancer Center Monoclonal Antibody Core Facility following established protocols [S1-S3]. Briefly, 6-week-old female BALB/c mice (Charles River Laboratories) were immunized with an *Aspergillus* *fumigatus* lysate (Miltenyi Biotech, #170-076-131) emulsified with adjuvants (Compete Freund and Incomplete Freund adjuvants at 1:1 ratio, BD Fico Adjuvants, #DF0638-60-7 and #DF0638-60-6). Mice received 5 injections of 20 µL into the footpad (one injection every 3 days). After the fifth injection, serum samples were obtained to confirm the presence of serum antibodies against the target (ELISA screening). Additional booster doses were administered as required. Popliteal lymph nodes from the immunized mice were harvested after 20 days and B-lymphocytes were fused with Sp2/0 myeloma cells to establish hybridomas. Hybridomas were selected using hypoxanthine-aminopterin-thymidine media. Screening for selection of positive clones against the *Aspergillus* lysates was performed by ELISA using bovine serum albumin (BSA) as negative control. The initially selected clones were then subcloned and re-screened by ELISA to select those with the highest affinity [S4].

ELISA screening

Costar EIA/RIA plates (Fisher Scientific) were coated with 0.1 µg/mL of *A. fumigatus* lysates or solvent (negative control) and allowed to dry overnight. Wells were blocked with phosphate-buffered saline (PBS) + 0.1% Tween 20 (PBST) containing 2% BSA for 1 h at room temperature (RT). Thereafter, culture supernatant from hybridoma plates (100 μL) or murine serum was added, and plates were incubated for 1 h at RT. Plates were washed thrice with PBST. Goat anti-mouse immunoglobulin G (IgG) Fc or anti-IgM with horseradish peroxidase (HRP) conjugate (100 μL; Jackson ImmunoResearch, Cat#115-035-071 and Cat#115-035-075) was then added and incubated for 1 h at RT. Plates were washed 5 times with PBST before the substrate was added. Absorbance was read at 450 nm.

Western blot analysis

One microgram of *A. fumigatus* lysates was loaded onto a 10% SDS gel, electrophoresed, and transferred to a nitrocellulose membrane. After blocking with 0.5% BSA in PBS for 1 h at RT, the membrane was incubated with AF-269-5 antibody (1:500 dilution in PBS + 0.5% BSA) for 1 h. After three washes with PBS, the blot was incubated with anti-mouse IgM-HRP conjugated secondary antibody and developed using a chemiluminescence kit (Bio-Rad, Cat# 1705062) according to the manufacturer’s protocol.

Fluorescence microscopy of AF-269-5 antibody-stained hyphae

Conidia/spores of various mold isolates and *Candida albicans* yeast cells (**Suppl. Table S1**) were seeded in 8-well chambered glass slides (500 cells per chamber) and incubated overnight in Roswell Park Memorial Institute (RPMI) medium supplemented with 10% fetal bovine serum (FBS). Plates were washed twice with PBS and blocked with 0.5% BSA in PBS (blocking buffer) for 1 h. Hyphae were then incubated with AF-269-5 antibody at 1:100 dilution in blocking buffer for 1 h. Chambers were washed thrice with PBS before addition of FITC-conjugated goat anti-mouse-IgM secondary antibody at 1:1000 dilution in blocking buffer. Slides were imaged using a fluorescence microscope (SP8, Leica Biosystems).

1. **Analysis of AF-CAR downstream signaling in Jurkat T cells**

## Culture of Jurkat T cells

Jurkat-Lucia *Nuclear factor of activated T-cells* (NFAT) reporter cells (InvivoGen, San Diego, USA) were cultured in RPMI medium 1640 supplemented with 2 mM Glutamax-1 (#35050-061, Life Technologies, Carlsbad, USA), 10% heat-inactivated FBS (Atlanta Biologics, Flowery Branch, USA), 100 U/mL penicillin, and 100 μg/mL streptomycin.

NFAT reporter assay

The AF-CAR was transduced to Jurkat-Lucia NFAT cells using a previously described lentiviral transduction method [S5]. AF-CAR expressing Jurkat-Lucia NFAT cells were used for downstream CAR signaling studies after stimulation with an *A. fumigatus* mycelial lysate (Miltenyi Biotec, Bergisch Gladbach, Germany). Upon NFAT activation, these cells secrete a coelenterazine-utilizing luciferase [S6]. Luciferase secretion into the cell culture supernatants was measured using the QUANTI-LUC^TM^ Lucia luciferase detection reagent (InvivoGen, San Diego, USA).

**Supplementary Figures**

**Supplementary Figure S1: AF-CAR T cells form clusters of activated cells at mature *A. fumigatus* mycelium.**

Representative micrographs showing binding and cluster formation of GFP^bright^ (strongly AF-CAR-expressing) cells at mature mycelium after 24 hours of co-culture. Scale: 200 µm in (**A**), 50 µm in (**B**).

**Supplementary Figure S2: The AF-CAR construct triggers downstream effector signaling in Jurkat T cells.**

(**A**) Flow cytometric evaluation of AF-CAR transduction efficiency in Jurkat T cells (percentage of CD3^+^ GFP^bright^ cells among all CD3^+^ cells). The lowest transduction efficiency (92%) of out 3 replicates is shown. (**B**) Baseline and *A. fumigatus* lysate (AfuLy)-induced luciferase activity as a surrogate of NFAT activation in Jurkat Lucia NFAT reporter cells. N = 3 independent replicates. RM one-way ANOVA with Tukey’s multiple comparison test. ** p < 0.01, *** p < 0.001. (**C**) Baseline, AfuLy-induced, and PMA/ionomycin-induced (positive control) CD69 expression on Control and AF-CAR-expressing Jurkat T cells after 18 hours of stimulation.

**Supplementary Figure S3: Monoclonal antibody AF-269-5 binds to caspofungin-pretreated *A. fumigatus* hyphae.**

Five hundred *A. fumigatus* AF-293 conidia were grown overnight in Roswell Park Memorial Institute medium supplemented with 10% fetal bovine serum and 0.25 µg/mL caspofungin (the minimum effective concentration of caspofungin against AF-293). The resulting hyphae, showing the typical short, hyper-branched phenotype after caspofungin treatment, were incubated with monoclonal antibody AF-269-5, washed, fluorescently labelled with FITC-tagged secondary antibody, and imaged microscopically as described in **Supplementary Methods**. Scale: 100 µm.

**Supplementary Figure S4: Schematic of the lentiviral vector (LV) for generation of AF-269-5-8a-CD137-CD3z CAR T cells.**

The full-length AF-CAR construct, inter splicing linker 2A, and GFP were subcloned into the third-generation self-inactivating LV vector dCAS9-VP64-GFP (Addgene, Water Town, USA) containing an EF1α promoter.

**Supplementary Figure S5: Gating strategy and representative dataset for flow cytometric analyses of AF-CAR T cells.**

(**A**) The lymphocyte population was identified by light scatter properties. (**B**) Dead cells (Amcyan^+^) were excluded. (**C**) Singlets were identified using forward scatter area (FSC-A) over height (FSC-H). (**D**) CD3^+^ T cells and CD3^+^ GFP^bright^ AF-CAR T cells were gated. (**E**) CD8^+^ cytotoxic T-cells were identified, and the remaining cells were considered T-helper cells. (**F-G**) Memory/effector subsets of T-helper cells (**F**) and cytotoxic T cells (**G**) were distinguished by CCR7 and CD45RA expression. Naïve T cells = CCR7^+^ CD45RA^+^, central memory T cells (T_CM_) = CCR7^+^ CD45RA^-^, effector memory T cells (T_EM_) = CCR7^-^ CD45RA^-^, terminally differentiated effector memory T cells with re-expression of CD45RA (T_EMRA_) = CCR7^-^ CD45RA^+^. Analyses shown in panels **E-G** were also applied specifically to CD3^+^ GFP^bright^ AF-CAR T cells (Q2 in **D**).

**Supplementary Movie S1: AF-CAR T cells target *A. fumigatus* hyphae and form clusters at mature mycelium.**

Movie composed of hourly time lapse micrographs capturing the first 30 h of co-culture of AF-CAR T cells and AF-293 GFP conidia at a 100:1 effector/target ratio. Note the accumulation of GFP^bright^ (strongly AF-CAR-expressing) cells at *A. fumigatus* hyphae over time.

**Supplementary Table S1: Fungal isolates used in this study.**

| **Species** | **Isolate identifier** | **Source** |
| --- | --- | --- |
| *Aspergillus fumigatus* | AF-293 | American Type Culture Collection |
| *Aspergillus fumigatus* | AF-293-GFP | Kind gift from Kieren Marr, Johns Hopkins  University School of Medicine, Baltimore, USA |
| *Aspergillus fumigatus* | AFIS-4931 | U.S. Centers for Disease Control and Prevention |
| *Aspergillus fumigatus* | AFIS-5341 | U.S. Centers for Disease Control and Prevention |
| *Aspergillus fumigatus* | AF-2793 | Clinical isolate from MD Anderson Cancer Center |
| *Aspergillus flavus* | AFL-120 | Clinical isolate from MD Anderson Cancer Center |
| *Aspergillus niger* | AN-102 | Clinical isolate from MD Anderson Cancer Center |
| *Aspergillus terreus* | AT-147 | Clinical isolate from MD Anderson Cancer Center |
| *Rhizopus arrhizus* | RA-749 | Clinical isolate from MD Anderson Cancer Center |
| *Mucor circinelloides* | MC-518 | Clinical isolate from MD Anderson Cancer Center |
| *Candida albicans* | Y-2515 | Clinical isolate from MD Anderson Cancer Center |

**Supplementary Table S2: Antibodies used for flow cytometry.**

| **Marker** | **Fluorochrome** | **Cat No.** | **Clone** | **Company** |
| --- | --- | --- | --- | --- |
| CD11b | APC-Cy7 | 560914 | ICRF44 | BD Pharmingen |
| CD11c | BV605 | 563929 | B-Ly6 | BD Horizon |
| CD3 | APC/BV786/PE | /563800/ | /SK-7/ | BD Biosciences |
| CD4 | APC-R-700/BV786 | 564976/563877 | RPA-T4/SK3 | BD Horizon |
| CD8 | Pacific Blue/APC-H7 | MHC-D0828/560179 | SK-1 | Invitrogen/  BD Biosciences |
| CD56 | BV786 | 564058 | NCAM-16.2 | BD Biosciences |
| CD45RA | PECy7 | 337167 | -- | BD Biosciences |
| CD62L | APC-Cy7 | 304814 | DREG-56 | BD Biosciences |
| CCR7 | PerCP-Cy5.5 | 561144 | 150503 | BD Biosciences |
| CD69 | APC-R-700 | 565154 | FN50 | BD Biosciences |

**Supplementary References**

[S1] J. Hu, L.T. Vien, X. Xia, L. Bover, and S. Li, Generation of a monoclonal antibody against the glycosylphosphatidylinositol-linked protein Rae-1 using genetically engineered tumor cells. Biol Proced Online 16 (2014) 3.

[S2] H. Qin, G. Wei, I. Sakamaki, Z. Dong, W.A. Cheng, D.L. Smith, F. Wen, H. Sun, K. Kim, S. Cha, L. Bover, S.S. Neelapu, and L.W. Kwak, Novel BAFF-Receptor Antibody to Natively Folded Recombinant Protein Eliminates Drug-Resistant Human B-cell Malignancies In Vivo. Clin Cancer Res 24 (2018) 1114-1123.

[S3] K.S. Voo, L. Bover, M.L. Harline, L.T. Vien, V. Facchinetti, K. Arima, L.W. Kwak, and Y.J. Liu, Antibodies targeting human OX40 expand effector T cells and block inducible and natural regulatory T cell function. J Immunol 191 (2013) 3641-50.

[S4] M. Uhlen, A. Bandrowski, S. Carr, A. Edwards, J. Ellenberg, E. Lundberg, D.L. Rimm, H. Rodriguez, T. Hiltke, M. Snyder, and T. Yamamoto, A proposal for validation of antibodies. Nat Methods 13 (2016) 823-7.

[S5] N. Anastasov, I. Hofig, S. Mall, A.M. Krackhardt, and C. Thirion, Optimized Lentiviral Transduction Protocols by Use of a Poloxamer Enhancer, Spinoculation, and scFv-Antibody Fusions to VSV-G. Methods Mol Biol 1448 (2016) 49-61.

[S6] Y.C. Kuo, C.F. Kuo, K. Jenkins, A.F. Hung, W.C. Chang, M. Park, B. Aguilar, R. Starr, J. Hibbard, C. Brown, and J.C. Williams, Antibody-based redirection of universal Fabrack-CAR T cells selectively kill antigen bearing tumor cells. J Immunother Cancer 10 (2022).
